# Molecular Vibrational Polariton Dynamics: What Can Polaritons Do?

**DOI:** 10.1021/acs.accounts.2c00796

**Published:** 2023-03-17

**Authors:** Wei Xiong

**Affiliations:** Department of Chemistry and Biochemistry, University of California, San Diego, 9500 Gilman Drive, La Jolla, California 92093-0358, United States

## Abstract

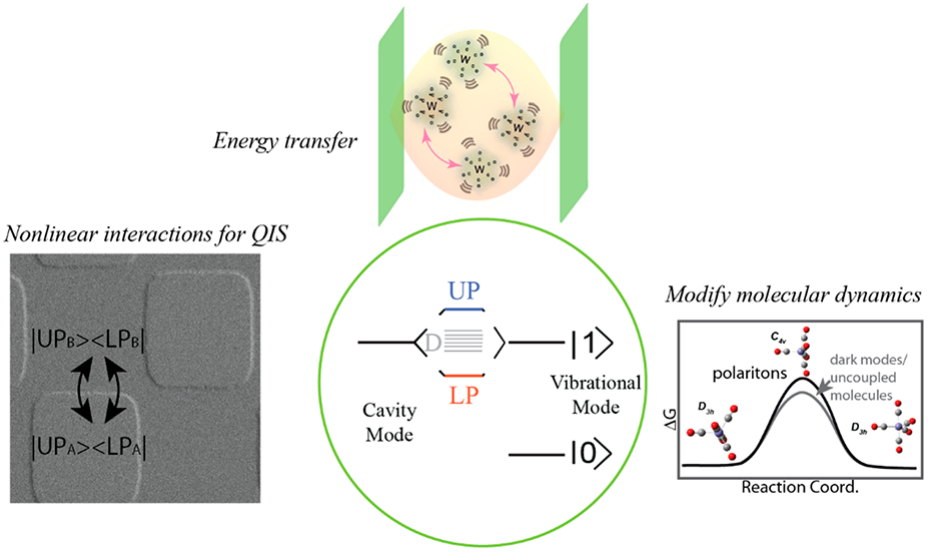

When molecular vibrational modes strongly couple to virtual states of photonic modes, new molecular vibrational polariton states are formed, along with a large population of dark reservoir modes. The polaritons are much like the bonding and antibonding molecular orbitals when atomic orbitals form molecular bonds, while the dark modes are like nonbonding orbitals. Because the polariton states are half-matter and half-light, whose energy is shifted from the parental states, polaritons are predicted to modify chemistry under thermally activated conditions, leading to an exciting and emerging field known as polariton chemistry that could potentially shift paradigms in chemistry. Despite several published results supporting this concept, the chemical physics and mechanism of polariton chemistry remain elusive. One reason for this challenge is that previous works cannot differentiate polaritons from dark modes. This limitation makes delineating the contributions to chemistry from polaritons and dark states difficult. However, this level of insight is critical for developing a solid mechanism for polariton chemistry to design and predict the outcome of strong coupling with any given reaction. My group addressed the challenge of differentiating the dynamics of polaritons and dark modes by ultrafast two-dimensional infrared (2D IR) spectroscopy. Specifically, (1) we found that polaritons can facilitate intra- and intermolecular vibrational energy transfer, opening a pathway to control vibrational energy flow in liquid-phase molecular systems, and (2) by studying a single-step isomerization event, we verified that indeed polaritons can modify chemical dynamics under strong coupling conditions, but in contrast, the dark modes behave like uncoupled molecules and do not change the dynamics. This finding confirmed the central concept of polariton chemistry: polaritons modify the potential energy landscape of reactions. The result also clarified the role of dark modes, which lays a critical foundation for designing cavities for future polariton chemistry. Aside from using 2D IR spectroscopy to study polariton chemistry, we also used the same technique to develop molecular polaritons into a potential quantum simulation platform. We demonstrated that polaritons have Rabi oscillations, and using a checkerboard cavity design, we showed that polaritons could have large nonlinearity across space. We further used the checkerboard polaritons to simulate coherence transfer and visualize it. A unidirectional coherence transfer was observed, indicating non-Hermitian dynamics. The highlighted efforts in this Account provide a solid understanding of the capability of polaritons for chemistry and quantum information science. I conclude this Account by discussing a few challenges for moving polariton chemistry toward being predictable and making the polariton quantum platform a complement to existing systems.

## Key References

- XiangB.; RibeiroR. F.; DunkelbergerA. D.; WangJ.; LiY.; SimpkinsB. S.; OwrutskyJ. C.; Yuen-ZhouJ.; XiongW.Two-Dimensional Infrared Spectroscopy of Vibrational PolaritonsProc. Natl. Acad. Sci. U. S. A.2018, 115, 4845–48502967444810.1073/pnas.1722063115PMC5948987.^1^*This work demonstrated that 2D IR spectroscopy can resolve polaritons and dark modes based on their distinct spectral features and verified that dark modes have optical strength due to chemical heterogeneity.*
- XiangB.; RibeiroR. F.; DuM.; ChenL.; YangZ.; WangJ.; Yuen-ZhouJ.; XiongW.Intermolecular Vibrational Energy Transfer Enabled by Microcavity Strong Light–Matter Coupling. Science2020, 368, 665–6673238172510.1126/science.aba3544.^2^*This work for the first time showed that by vibrational strong coupling (VSC) to photonic cavity modes, polaritons could enable vibrational energy transfer between different molecules.*
- ChenT.; DuM.; YangZ.; Yuen-ZhouJ.; XiongW.Cavity-Enabled Enhancement of Ultrafast Intramolecular Vibrational Redistribution over Pseudorotation. Science2022, 378, 790–7943639524110.1126/science.add0276.^3^*This work unambiguously demonstrated that under VSC, polaritons can modify single-step barrier crossing dynamics by suppressing it and promoting an alternative intramolecular vibrational redistribution channel, whereas the dark modes did not change the dynamics.*
- XiangB.; WangJ.; YangZ.; XiongW.Nonlinear Infrared Polaritonic Interaction between Cavities Mediated by Molecular Vibrations at Ultrafast Time Scale. Sci. Adv.2021, 7, eabf63973396294910.1126/sciadv.abf6397PMC8104880.^4^*This work showed that photon hopping can enable nonlinear interactions of polaritons with each other across space, enabling its application for future quantum information technologies.*

## Motivations and Background

When molecular modes strongly couple to photonic cavity modes, such that the molecules exchange energy with photons at higher rates than their corresponding dissipation, the light–matter interactions are in the strong coupling regime, forming so-called polaritons.^5−7^ While it is an established concept in quantum electrodynamics, the polariton concept has recently caught the attention of chemistry. Ebbesen pioneered this concept that when molecular vibrational modes are strongly coupled with the cavity modes and form molecular vibrational polaritons (MVPs), they can modify chemistry^8−10^ because the polaritons have different energy levels and wave functions from the composing vibrational modes. This novel concept of modifying chemical events by vibrational strong coupling (VSC) was supported by several research results using multiple techniques, including IR, UV–vis, and mass spectrometry,^11−14^ which have led to an exciting emerging new field termed polariton chemistry.

**Figure 1:**
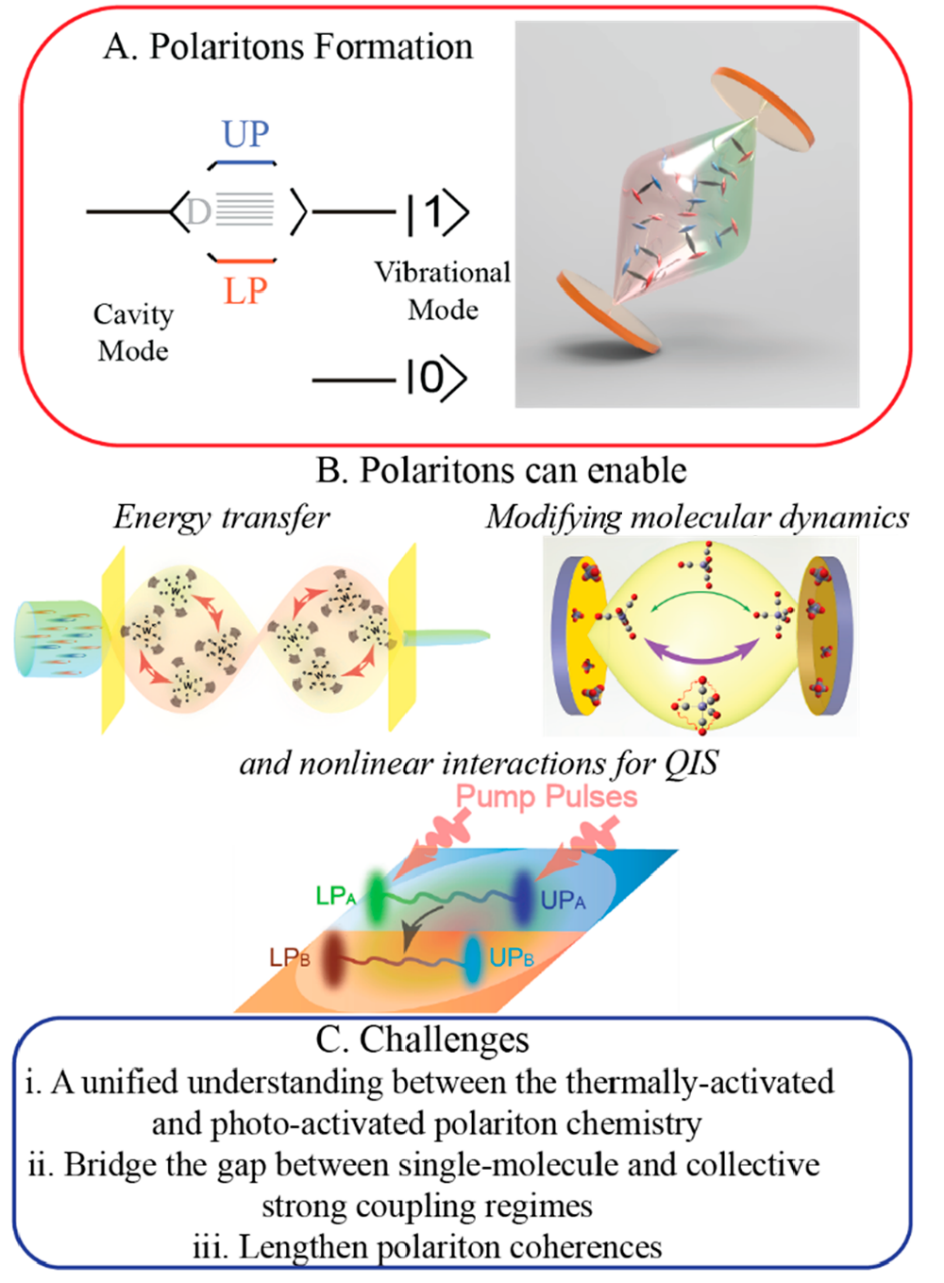
Molecular vibrational polaritons, what they can do, and a few remaining challenges. (A) Energy diagram of polaritons (UP and LP) and dark modes (D) under the collective VSC regime. (B) Chemical physics enabled by polaritons. (C) A few challenges in this field.

Given the great promise of polariton chemistry, it is necessary to have a clear mechanism of polariton chemistry that allows predictions of whether VSC can influence a given reaction and eventually leads to rational designs of photonic cavities to catalyze specific reactions.^15,16^ The understanding of polariton chemistry is still not fully developed, partly because it is new and partly because of a fundamental gap between experiment and theory, which I explain below. Because the dipolar interactions between a single molecule and the cavity mode (*g*_0_) are often not strong enough, it requires an ensemble of *N* molecules to interact with the cavity mode and reach VSC collectively. The collective coupling strength is proportional to , and it requires *N* ≈ 10^10^ to reach the so-called collective VSC regime. The consequence of the collective VSC is that there are also *N* – 1 dark modes that have no photonic components in their wave functions^10,16−19^ and resemble localized (or semilocalized^20−23^) molecular modes (Figure 1A). Thus, dark modes are not expected to influence chemical processes. Because the *N* – 1 dark modes significantly outnumber the two polariton modes, chemical reactions should be dictated by dark modes, thereby leading to little changes to reactions. Indeed, several theoretical works on the collective VSC regime did observe little changes to chemical reactions,^24−30^ and a couple of experimental works reported null results.^31,32^ On the other hand, theoretical works on the single-molecule VSC regime,^33−36^ i.e., making a single molecule strongly coupled to the cavity modes, provided unambiguous evidence to support that polaritons can modify reactions (and there were no dark modes in this case), agreeing with some experiments in the collective VSC regime.^11−14^ Given the myriad of evidence supporting polariton chemistry in the collective coupling regime (despite a few reports on issues of reproducibility^31,32^) and the unavoidable influence of dominating dark modes from the theory perspective, it is critical to answer the question of how the interplays between polaritons and dark modes can lead to polariton chemistry. Pertinent questions include the following: What can polaritons do? Can dark modes change chemistry? How does VSC influence the vibrational energy dynamics to affect reactions? This challenge emphasizes the importance of differentiating polaritons from dark modes and following their dynamics to understand the roles of polaritons and dark modes in modifying chemistry. In this Account, I will discuss ultrafast polariton dynamic research from my group and highlight what we have learned about the capabilities of polaritons (Figure 1B) and conclude with an outlook of remaining challenges (Figure 1C).

## 2D IR Spectroscopy for Vibrational Polaritons

Two-dimensional infrared (2D IR) spectroscopy,^37−48^ a coherent multidimensional ultrafast nonlinear optical technique, is well-suited for differentiating polaritons and dark reservoir modes. After the first report of the ultrafast IR pump–probe study of MVPs by Dunkelberger, Owrutsky, and co-workers^49^ and inspired by theoretical works,^50^ our group collaborated with the Naval Research Laboratory group to implement 2D IR spectroscopy to study polariton dynamics.^1,51^ We showed that 2D IR spectroscopy has unique capabilities to resolve dark modes from polariton states. This method has since been adopted to study pure polaritonic responses and polaritons in open cavity systems.^52−54^

**Figure 2:**
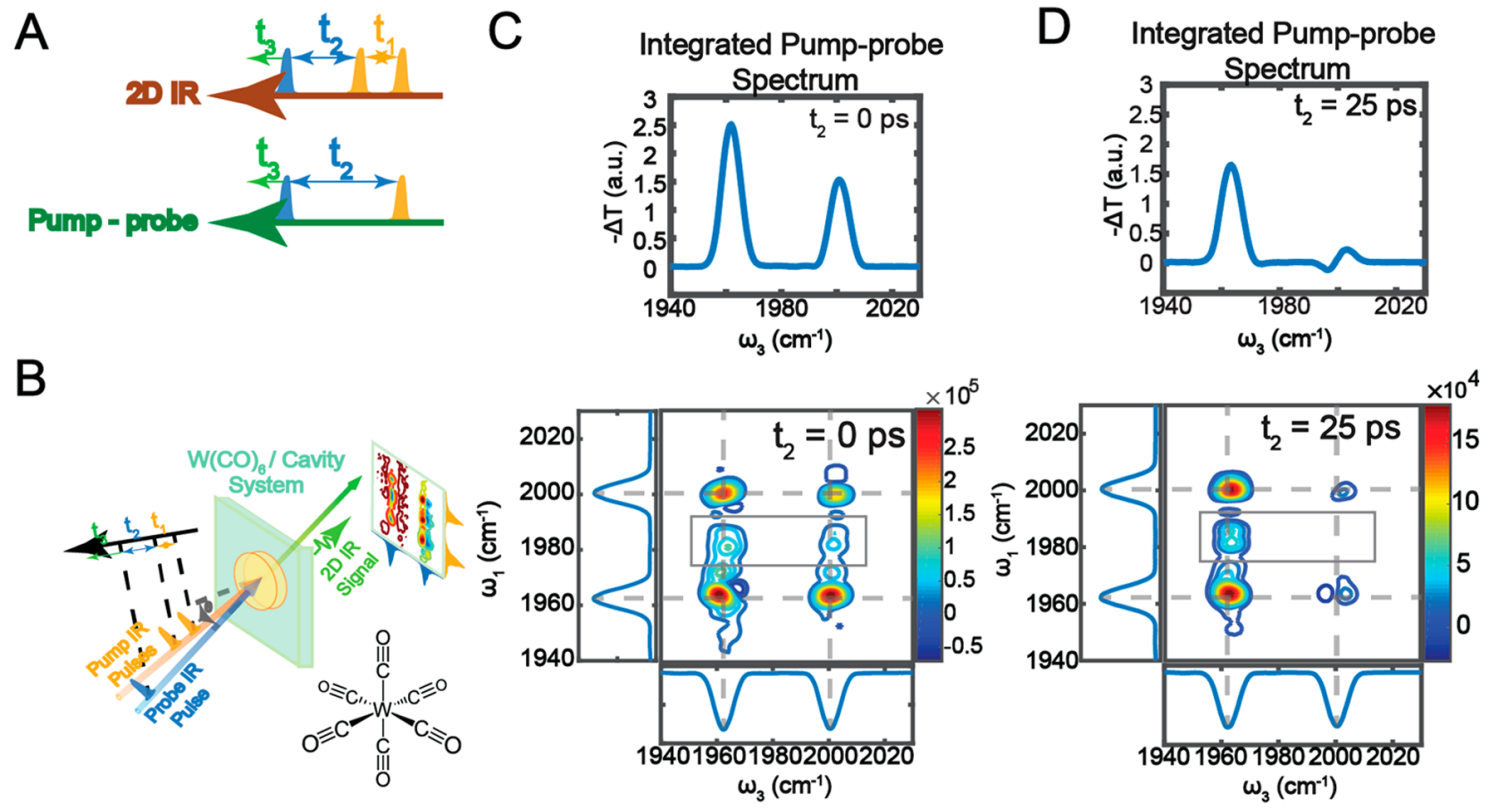
2D IR spectroscopy of MVPs. (A) Pulse sequences of 2D IR and pump–probe spectroscopy. (B) 2D IR experimental setup illustration. We used a pump–probe geometry where the first two pulses are collinear and compose the pump beam and the third pulse acts as the probe beam and the local oscillator. The pump and probe beams approach the samples at the same incident angles to study polaritons with the same energy. (C) (top) At a short *t*_2_ time delay, the pump–probe spectrum shows absorptive features due to polaritonic nonlinear interactions. (bottom) The 2D IR spectrum shows similar absorptive features but clearly shows cross-peaks (at the antidiagonal corner of the dashed box), indicating polariton–polariton interactions. (D) (top) At a long *t*_2_ time delay, the pump–probe spectrum evolves to a derivative shape at ω_3_= ω_UP_ because of Rabi splitting contraction and an absorptive feature at ω_3_= ω_LP_ due to the strong 1 → 2 transitions of dark reservoir modes. (bottom) The corresponding 2D IR spectrum shows similar features. It is noticeable that there are peaks of dark modes at ω_1_ = ω_dark_ in the 2D IR spectra (gray boxes in the bottom spectra of C and D). Thus, 2D IR spectroscopy can differentiate polaritons and dark modes. (B) and (D) are adapted with permission from ref (1). Copyright 2018 the authors of ref (1), under exclusive license to the National Academy of Sciences. (C) is from ref (51). CC BY-NC 4.0.

2D IR spectroscopy uses a three-pulse pulse sequence to probe the evolution of molecular vibrational energy in the system (Figure 2A). The first pulse prepares the system into a vibrational coherence state that is converted to either a population or coherence state by the second pulse. After waiting for a certain time delay (*t*_2_), the third pulse (probe) relaunches the vibrational coherences, emitting an IR signal. The IR signal is heterodyned by a local oscillator pulse (often the same third pulse), dispersed by a spectrograph, and detected by a mercury–cadmium–telluride array detector. The measured IR spectrum (whose axis is noted as ω_probe_ or ω_3_) encodes the latter coherence dynamics along *t*_3_ after the third IR pulse interaction. We scan the time delay between the first and second IR pulses (*t*_1_) and record the corresponding pump–probe spectrum to characterize the first vibrational coherence dynamics. The *t*_1_ series pump–probe dynamics is Fourier transformed into a spectrum along ω_pump_ or ω_1_. Thus, the correlation between ω_pump_ and ω_probe_ can be plotted as a 2D map, known as a 2D IR spectrum. One advantage of 2D IR spectroscopy versus a pump–probe experiment is that molecular resonances are distinguished along ω_pump_. Thus, it can differentiate the initial quantum states and follow their evolutions by scanning the delay time *t*_2_.

We showed that 2D IR spectroscopy has an advantage of differentiate various quantum states on polariton systems (Figure 2B). Below, I use the polaritons created by the asymmetric vibrational modes of W(CO)_6_ under VSC as an example. The 2D IR spectrum showed two clear peaks along the diagonal, representing the UP and LP resonances, and cross-peaks indicating the polaritons interacting with each other (Figure 2C,D, bottom). However, the more striking feature (gray boxes) is that there are peaks at ω_1_ = ω_dark_, suggesting that the dark modes are also excited and interacting with polaritons.^1^ The dark modes are visible because of the chemical inhomogeneity, as theory predicted.^55^ Nevertheless, the fact that the 2D IR spectrum has distinct polariton and dark mode features along ω_1_ gives it a unique strength to address the existing challenges in polariton chemistry.

The 2D IR dynamics of MVPs differ from a pure molecular system, as the polaritons are hybrids between molecules and photons. When *t*_2_ is shorter than the lifetime of polaritons (*t*_polariton_), the polariton populations and their coherent nonlinear interactions dominate, leading to the 2D IR and pump–probe peaks with pure absorptive line shape (Figure 2C).^51^ We have attributed the nonlinearity to nonlinear dephasing, and further theoretical studies are necessary for this mechanism. However, the mechanism is well-understood when *t*_2_ is longer than the lifetime of the polaritons. In this time regime, polaritons decay into the dark reservoir modes. Thus, the dark reservoir modes are excited from the ground state, effectively reducing the population of molecules to couple to the cavity modes, further decreasing the collective coupling strength and, thereby, the Rabi splitting. As a result, the UP peak frequency shifts down and the LP peak frequency shifts up, leading to derivative features in the 2D IR spectrum. The derivative features are difficult to resolve in the ω_3_ = ω_LP_ region because they are overwhelmed by the large absorptive feature from the *v* = 1 → 2 transition of the dark reservoir modes due to its anharmonicity (Figure 2D).^1,18,49,56^ Overall, the early-time coherent dynamics of polariton states (*t*_2_ < *t*_polariton_) can be useful for potential quantum applications. In contrast, the dynamics in the late-time regime (*t*_2_ > *t*_polariton_) corresponds to the incoherent population dynamics of molecules that participated in polariton formation. This incoherent dynamics reveals how the excited polariton energy is deposited into pure molecular vibrational modes. Because this deposited energy can stay in molecular modes for hundreds of picoseconds, this process could thereby influence chemical reactions. Below, I will mostly focus on the late-time incoherent dynamics related to polariton chemistry and then use one section to discuss our efforts to develop MVPs into a potential quantum simulation platform.

## MVP-Mediated Vibrational Energy Transfer

**Figure 3:**
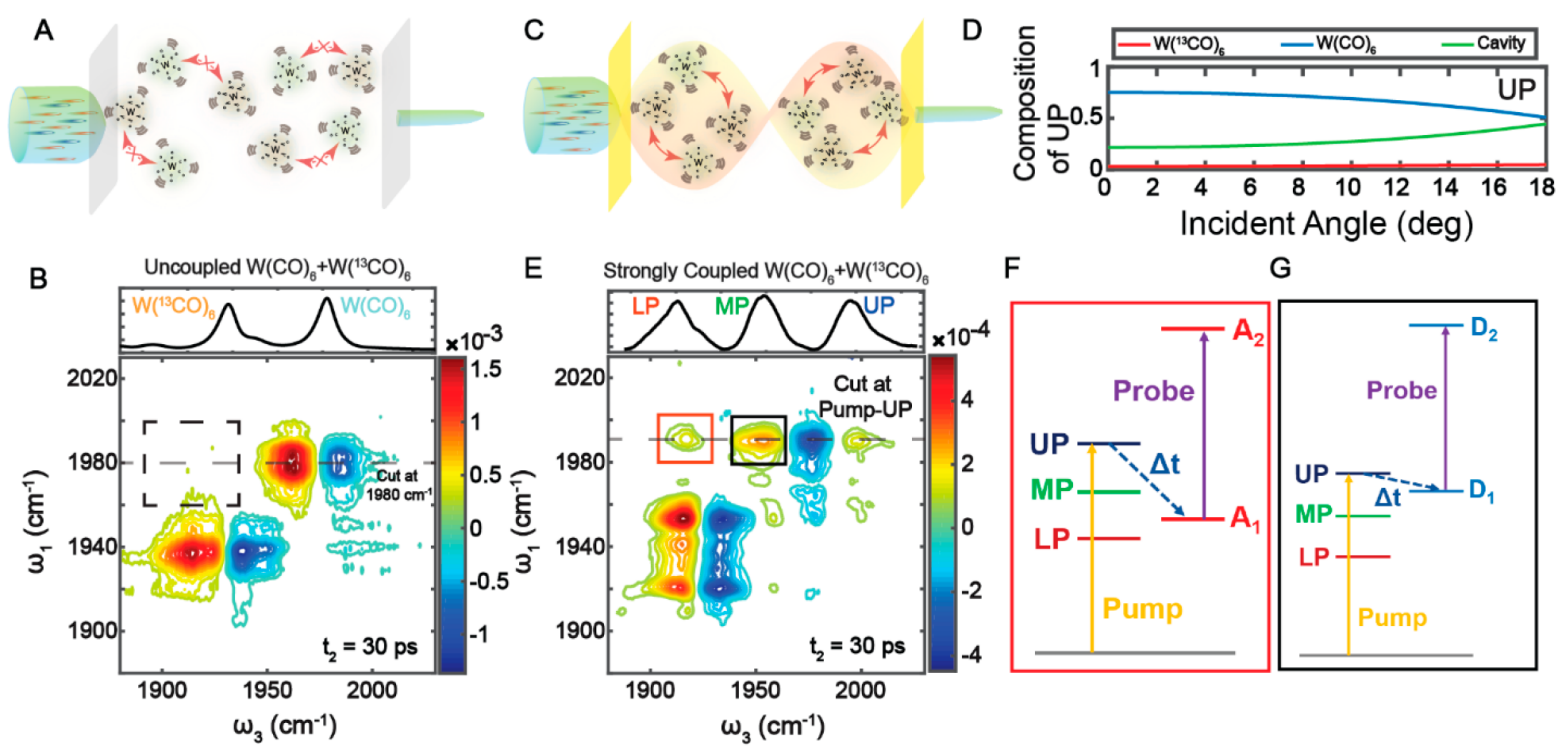
Polariton-enabled intermolecular vibrational energy transfer. (A) Under normal circumstances, there is no directional VET from W(CO)_6_ and W(^13^CO)_6_ because it is outcompeted by energy dissipation to the solvents. (B) The absence of intermolecular VET is confirmed in the 2D IR spectrum, as there is no cross-peak between the two species. (C) By VSC, intermolecular VET is enabled. (D) The UP is composed mostly of W(CO)_6_ (donor, D) and cavity modes, with little contribution from W(^13^CO)_6_ (acceptor, A). (E) In the 2D IR spectrum, when UP is excited, a clear cross-peak appears at ω_3_ = ω_LP_ (orange box), indicating that exciting UP can transfer energy to A, as shown in (F), while the cross-peak at ω_3_ = ω_MP_ (black box) reports the energy relaxation to the D channel, as shown in (G). The intensity ratio between the peaks at ω_3_ = ω_LP_ and ω_3_ = ω_MP_ suggests that intermolecular VET occurred. (A–E) are adapted with permission from ref (2). Copyright 2020 the authors of ref (2), under exclusive license to the American Association for the Advancement of Science.

The photonic component of polaritons grants them delocalization. As a result, it has been shown in the exciton–polariton regime that excitonic energy can be delocalized among spatially separated films.^57,58^ It is natural to consider whether such a polariton-mediated energy transfer could happen to vibrational degrees of freedom. Compared to excitons, the dipole strengths of vibrational modes are much smaller, which makes the dipole–dipole coupling-mediated energy transfer negligible (with the exceptions of a few special systems^59,60^) (Figure 3A). The lack of intermolecular vibrational energy transfer (VET) is evidenced by the absence of cross-peaks in the 2D IR spectrum of the W(CO)_6_ and W(^13^CO)_6_ mixtures outside the cavity (dashed box area in Figure 3B). However, controlling VET is critical in manipulating the vibrational energy flow for chemical transformation^61^ and transducing signals among biological entities.^62^ Thus, it would be an important advance if energy transfer among specific vibrational modes could be turned on through engineering of VSC (Figure 3C), which would enable many new applications.

To realize it, we tuned the cavity so that its resonance is between the asymmetric vibrational modes of W(CO)_6_ and W(^13^CO)_6_, leading to a three-polariton system.^2,63−65^ We ensured that the coupling strength was high enough that the UP, MP, and LP were composed of the two vibrational modes and the single cavity mode, as indicated by their Hopfield coefficients. Noticeably, the UP was primarily composed of W(CO)_6_ and the cavity mode, with a small contribution from W(^13^CO)_6_ (Figure 3D). The molecular compositions of LP were flipped compared to UP.

In the 2D IR spectrum, at long *t*_2_, such as *t*_2_ = 30 ps, a clear cross-peak appeared at ω_1_ = ω_UP_, ω_3_ = ω_LP_ (orange box in Figure 3E). Because the data were taken long after the polariton relaxed to the dark reservoir modes, this cross-peak indicated that when UP was excited, its energy could be transferred to the first excited state of W(^13^CO)_6_, whose *v* = 1 → 2 transition was resonant with LP transitions. The excited population of W(^13^CO)_6_ was 6 times more than expected if the cross-peak was solely due to the W(^13^CO)_6_ compositions in the wave functions of UP. Thus, this result indicated that VSC enabled a significant energy transfer from W(CO)_6_ to W(^13^CO)_6_. Furthermore, the efficiency of VET could be enhanced by increasing the cavity thickness, which intuitively was because the long cavity allowed more cavity (and polariton) lifetime to mediate energy transfer. This intermolecular VET occurred only as a downhill energy flow, whereas the uphill process was missing. It was somewhat surprising as the energy difference between polaritons was close to *k*_B_*T*. The downhill process could be due to favorable population transfer from UP to a manifold of MP states, which remains to be further studied. Nevertheless, the preferred downhill transfer should be considered for future designs of polariton-mediated energy transfer process.

## Vibrational-Polariton-Modified Pseudorotation Dynamics

A central question in polariton chemistry research involves the roles of polaritons and dark modes in modifying chemical reactions. The question comes from the prediction that polaritons can change the potential energy landscapes of reactions and that dark modes behave like regular molecules.^16^ However, because of the large density of states of the dark reservoir modes in the collective VSC regime, the observation of modified chemistry in collective VSC implies that dark modes must also influence chemical transformations. There are a couple of factors contributing to the present challenge. First, most techniques implemented for studying polariton chemistry to date only monitor the end products without differentiating whether the initial states were polaritons or dark modes. Second, nearly all reactions studied were multiple-step complex reactions,^66^ making it difficult to pinpoint the mechanisms.

**Figure 4:**
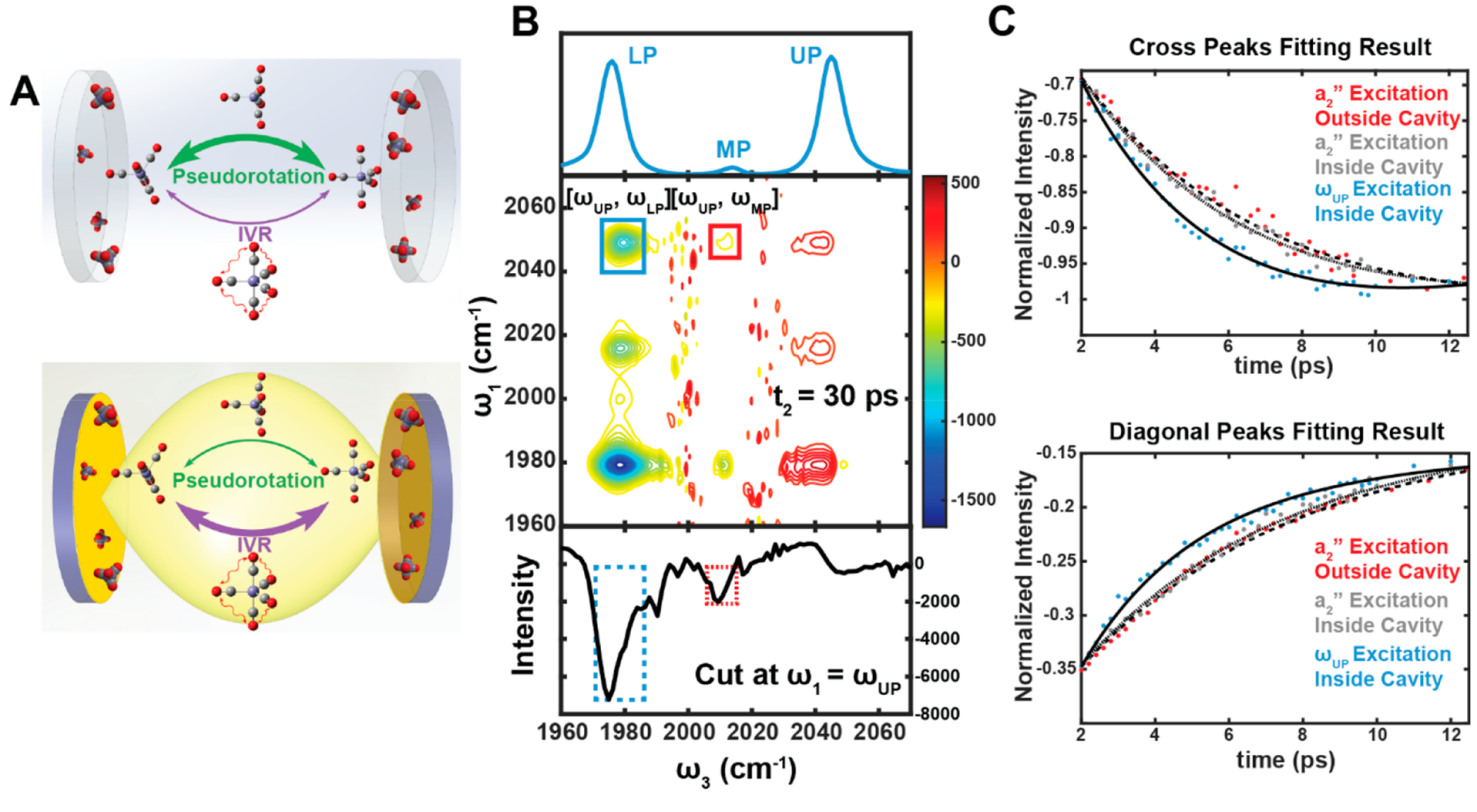
Polariton-modified chemical dynamics. (A) Under normal conditions, the Fe(CO)_5_ molecules favor pseudorotation over IVR (top), but under VSC, polaritons accelerate IVR and slow down pseudorotation. (B) The 2D IR spectrum of polaritons (middle) is formed by strong coupling of Fe(CO)_5_ with the cavity modes. The [ω_UP_, ω_LP_] (cross-peaks, labeled by a blue square) and [ω_UP_, ω_MP_] (diagonal peaks, red square) are used to follow the energy exchange between the a_2_^″^ and e′ modes. (top) Linear IR spectrum and (bottom) spectral cut at ω_1_ = ω_UP_ of the system. (C) Energy exchange dynamics and fits show that under VSC, exciting UP accelerates the energy exchange dynamics (blue), while when the dark a_2_^″^ modes are excited (gray), the energy exchange remains similar to that outside of cavities (red). Adapted with permission from ref (3). Copyright 2022 the authors of ref (3), under exclusive license to the American Association for the Advancement of Science.

My group addressed this challenge by focusing on a single-step chemical transformation, the well-characterized Berry pseudorotation of Fe(CO)_5_,^67^ using 2D IR spectroscopy, by which we could differentiate the polariton-initiated pseudorotation dynamics from that initiated by dark reservoir modes. The pseudorotation of Fe(CO)_5_ is an isomerization where the axial and equatorial CO ligands exchange with each other, making the products identical to the reactants after the dynamics. This isomerization also leads to energy exchange between the corresponding axial (a_2_^″^) and equatorial (e′) modes, which is manifested as a cross-peak in the 2D IR spectrum. However, an intramolecular vibrational redistribution (IVR) channel could also be a minor pathway (Figure 4A, top). Harris, Cahoon, and co-workers have characterized its dynamics outside of cavities.^38^

When strongly coupled to the cavity modes, the a_2_^″^ and e′ modes split into three polariton states (UP, MP, and LP), with UP composed mostly by the a_2_^″^ mode, LP by the e′ mode, and MP as a mixture of the two. Using the cross-peaks at ω_3_ = ω_LP_, we followed the energy exchange dynamics initiated by pumping UP and dark a_2_^″^ modes (Figure 4B). When UP was pumped, the energy exchange rate (*k*_ex_) was 0.113 ± 0.009 ps^–1^, which is higher than the counterpart of Fe(CO)_5_ outside of the cavity (*k*_ex_ = 0.084 ± 0.002 ps^–1^); in contrast, when dark a_2_^″^ modes were pumped, *k*_ex_ = 0.090 ± 0.006 ps^–1^, similar the case of outside of the cavity. Thus, it appeared that under VSC, only polaritons could modify the dynamics, while dark modes could not (Figure 4C).

**Figure 5:**
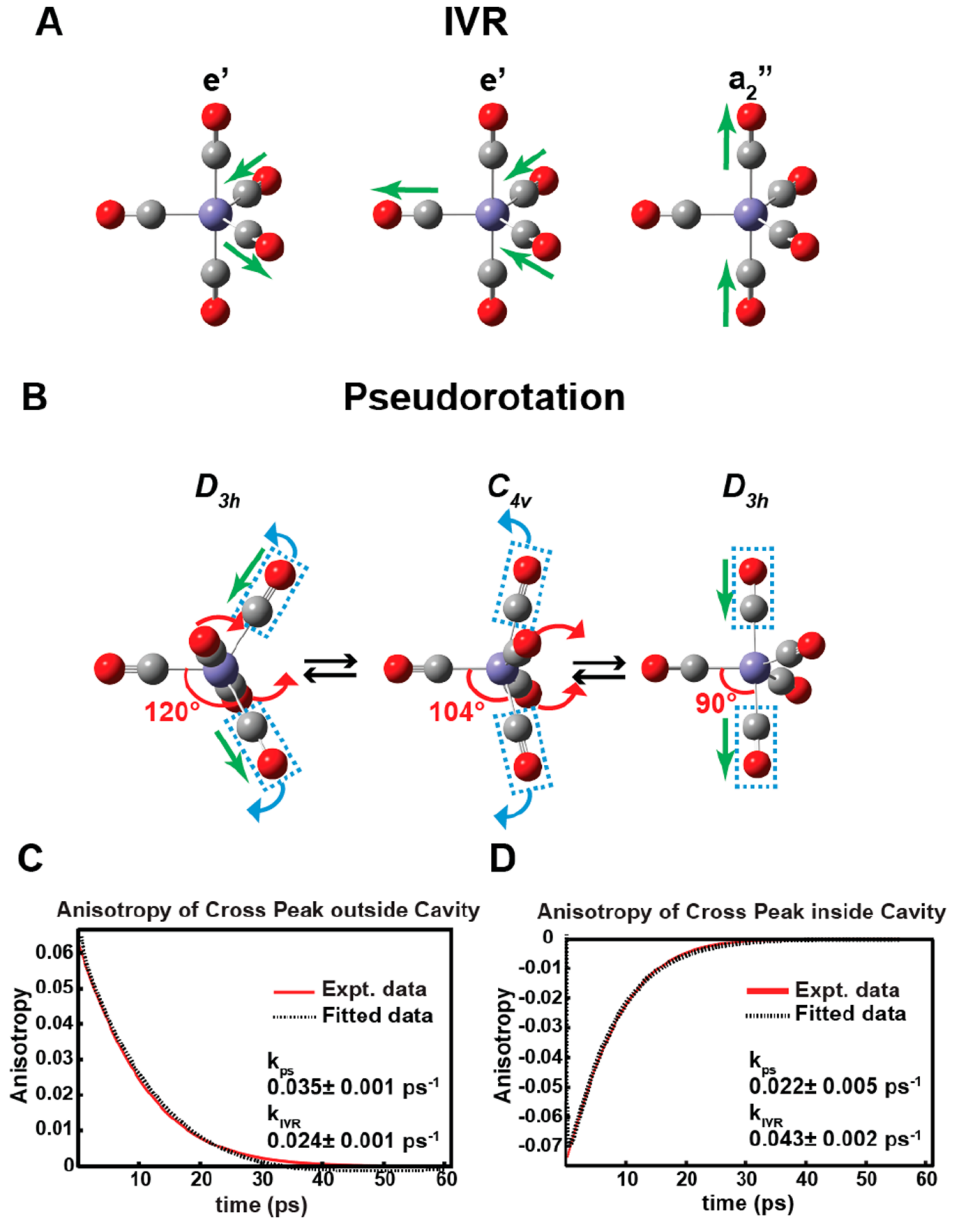
Anisotropy of energy exchange dynamics. (A) Depiction of the eigenvectors for the a_2_^″^ and doubly degenerate e′ vibrational modes of Fe(CO)_5_. IVR leads to energy transfer between modes perpendicular to each other, leading to an initial anisotropy of 0.4. (B) When the energy exchange is through pseudorotation, the energy transfer is between two dipoles parallel to each other, resulting in an initial anisotropy of −0.2. (C) The anisotropy of the energy exchange cross-peak indicates that the dynamics is dominated by pseudorotation outside of the cavity. (D) Under VSC, the anisotropy indicates that exciting UP accelerates IVR and suppresses pseudorotation. Adapted with permission from ref (3). Copyright 2022 the authors of ref (3), under exclusive license to the American Association for the Advancement of Science.

However, the dynamics that caused the acceleration of energy exchange deserved more attention because other channels, such as IVR, could also lead to energy exchange between the a_2_^″^ and e′ modes and create the same cross-peaks. We differentiated IVR and pseudorotation by their initial anisotropy (Figure 5).^68^ When IVR occurred, the energy exchange happened between two normal modes perpendicular to each other, resulting in an initial anisotropy of −0.2 (Figure 5A). When pseudorotation occurred, the a_2_^″^ mode morphed into the e′ mode without changing the orientation of the modes in the molecular frame, leading energy exchange between two modes parallel to each other. In this case, the initial anisotropy should be 0.4 (Figure 5B). The actual anisotropy when pumping UP was −0.08, and the outside cavity case was 0.06. Thus, qualitatively, we concluded that under VSC, it indeed promoted IVR to dominate over pseudorotation and accelerated the overall energy exchange. Quantitative fitting using kinetic models led to *k*_ps_ = 0.022 ± 0.005 ps^–1^ and *k*_IVR_ = 0.043 ± 0.002 ps^–1^ when UP was excited, compared to *k*_ps_ = 0.035 ± 0.001 ps^–1^ and *k*_IVR_ = 0.024 ± 0.001 ps^–1^ when the molecules were outside of the cavity (Figure 5C,D). Thus, VSC not only promoted IVR but also decelerated pseudorotation compared to the case without VSC. However, dark modes did not have such an effect. The reflectivity of distributed Bragg reflector (DBR) optics can depend on the polarizations of incoming beams, which may influence the anisotropy. In this experiment, such an effect was small, as the anisotropy of diagonal peaks remained close to the theoretical value of 0.4. Clearly, polaritons in VSC can promote intramolecular or intermolecular VET. The physical origin of suppressing pseudorotation could be loss of its driving force due to IVR or being hindered by the excited phonons. Other driving forces, such as excitation to hot vibrational modes,^69^ could also play a role. This VSC-modified dynamics was just recently supported by a hybrid quantum-mechanical/molecular-mechanical cavity molecular dynamics scheme,^70^ which could provide critical mechanistic insights in the future. The reflectivity of distributed Bragg reflector (DBR) optics can depend on the polarizations of incoming beams, which may influence the anisotropy. In this experiment, such an effect was small, as the anisotropy of diagonal peaks remained close to the theoretical value of 0.4.

This work unambiguously demonstrated that polaritons can influence chemical transformations—the central concept in polariton chemistry—but also clarified that the dark modes behave like uncoupled molecules. Thus, it added a pillar to the polariton chemistry research and pointed out that future enhancement of polariton chemistry could lie in miniaturizing the cavity volume to reduce dark modes.^71−73^

## Toward Vibrational-Polariton-Based Quantum Technology

**Figure 6:**
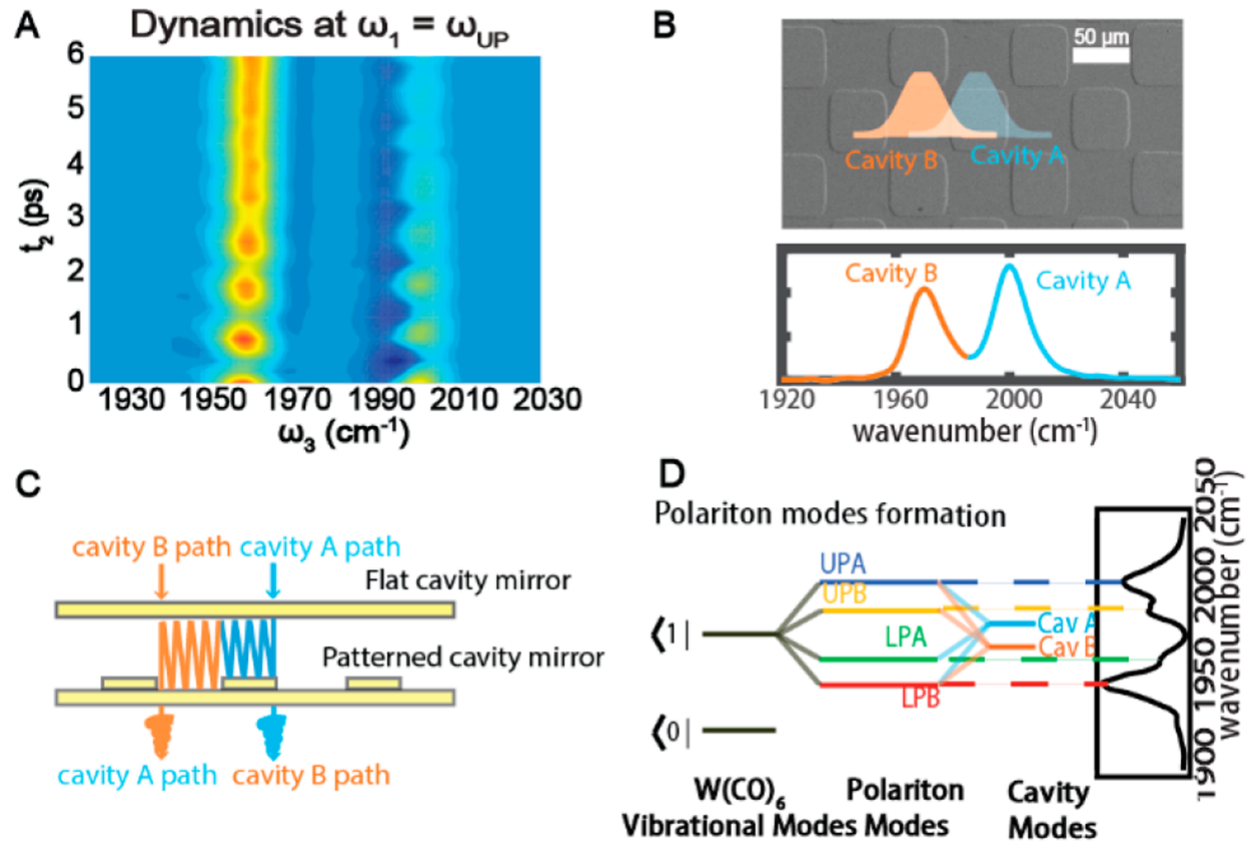
Polariton coherence and the checkerboard cavity. (A) UP and LP oscillate at the Rabi frequency. (B) A pair of cavity modes are created by preparing square-shaped extrusions on the cavity optics, forming a checkerboard pattern. (C) The checkerboard pattern optic and a flat mirror can form cavities with different resonant frequencies that spatially overlap through evanescent waves. (D) When strongly coupled with the asymmetric mode of W(CO)_6_, each cavity forms its own UP and LP. The linear spectrum of the system is shown at the right. From ref (4). CC BY-NC 4.0.

While strong coupling to photons can change molecular properties, VSC can also modify photonic properties. One direct result is the strong optical nonlinearities of photons that otherwise would not exist. The strong optical nonlinearity could enable the use of photons/polaritons as quantum bits (qubits). My group has shown that nonlinearity can be modulated by simply controlling the cavity size. More importantly, we observed Rabi oscillations between LP and UP (Figure 6A).^51,74^ The Rabi oscillation only lasted for about 5 ps, as determined by the Q factor of the cavity. However, it indicated that coherence between LP and UP could be prepared and act as a qubit.

We have made a few steps to prepare vibrational polaritons as a new quantum technology platform by showing that polaritons can nonlinearly interact with each other when sitting in different cavities.^4,75^ We used photolithography and deposition techniques to create a checkerboard-pattern DBR, with adjacent squares having an ∼200 nm height difference. Thus, when the patterned DBR was paired with a flat DBR, they formed neighboring cavities with different longitudinal thicknesses and resonant frequencies (Figure 6B,C). Each cavity could form its own UP and LP with W(CO)_6_, resulting in four polariton peaks in the linear spectrum (Figure 6D).

**Figure 7:**
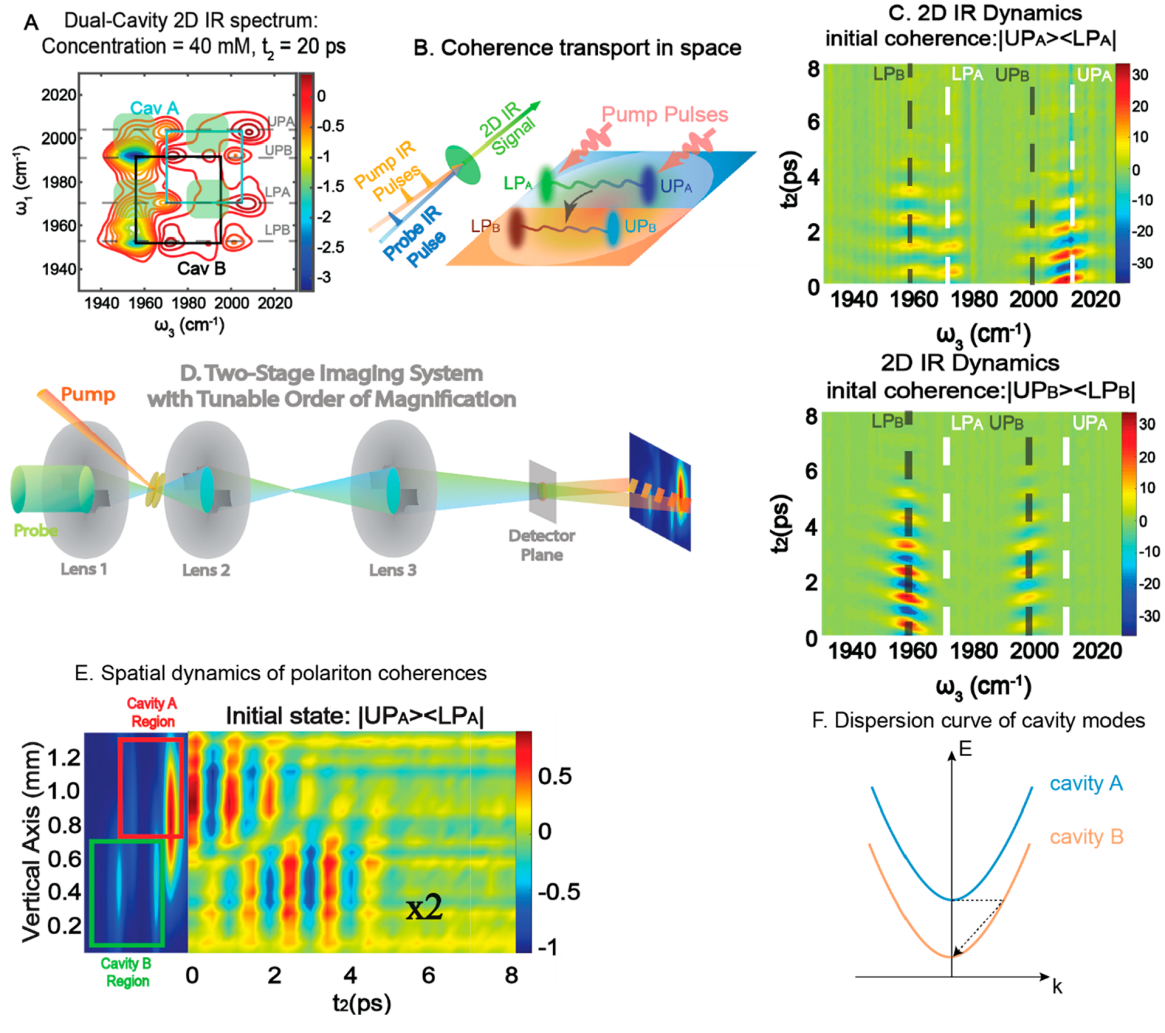
Intercavity polaritonic interactions and coherence transfer. (A) The 2D IR spectrum has cross-peaks (labeled by green shades), indicating that when polaritons in cavity A (Figure 6B) are excited, they also perturb polaritons in cavity B. (B) Schematic of the coherence transport experiment using the checkerboard polariton platform. The shaped IR pulses prepare polariton coherences in cavity A, and the coherences then are transferred to B and are followed by probe pulses. (C) Unidirectional coherence transfer is seen when cavity A coherences, such as |UP_A_⟩⟨LP_A_|, are transferred to cavity B and trigger coherence oscillations of polaritons in cavity B (top). The same coherence transfer does not happen when coherences, such as |UP⟩⟨LP_B_|, are prepared in cavity B (bottom). (D) 2D IR imaging setup allows resolution of the spectral peak frequency along the horizontal direction and the spatial location of polaritons along the vertical direction. (E) Ultrafast spectral image showing that after |UP_A_⟩⟨LP_A_| is prepared in cavity A, it is indeed transferred to polaritons in cavity B across space. (F) The unidirectional coherence transfer can be explained by the cavity dispersion curve. Cavity mode A always has higher energy than cavity mode B at the same momentum. Thus, photons in A can scatter to cavity B with a large momentum but the same energy and then relax to the bottom of the dispersion curve. The opposite is less favorable due to the energy penalty. (A) is from ref (4). CC BY-NC 4.0. (B–E) are adapted with permission from ref (76). Copyright 2021 Wiley-VCH.

Using 2D IR spectroscopy, we resolved cross-peaks indicating that when the polaritons in cavity A were excited, the polaritons in cavity B responded to the excitation (green shaded area in Figure 7A). Thus, nonlinear interactions exist between polaritons in different cavities. The existence of nonlinearity across space arises because of the photonic cavity evanescent wave and the large molecular nonlinearity.

We used this platform to simulate and visualize coherence transfer between cavities.^76^ Coherence transfer has been considered important in the energy relay of biological and chemical systems.^77,78^ The natural spatial (nanometers) and temporal (femtosecond) scales of coherence transfer made it difficult to resolve simultaneously in both domains. The checkerboard polariton platform, whose cavity lateral separation was 50 μm, made it possible to excite coherence or population in one cavity and watch it evolve in space and time (Figure 7B). We spatially visualized the polariton signals by converting the 2D IR spectrometer into a spectromicroscope that could resolve the spectra along the horizontal dimension of the detector and the spatial location of the signals vertically (Figure 7D). This new instrument thus allowed the coherence dynamics to be tracked in spatial, temporal, and frequency domains.

When the |UP_A_⟩⟨LP_A_| coherence was prepared in cavity A, it launched Rabi oscillations in spectral peaks located at ω_3_ = ω_UPA_ or ω_LPA_, but quickly the oscillations also appeared in peaks at ω_3_ = ω_UPB_ or ω_LPB_. This result indicated that coherence from cavity A was transferred to cavity B (Figure 7C, top). The spatial–temporal plot confirmed this conclusion. The Rabi oscillation at ω_3_ = ω_UPA_ was initially launched in cavity A and quickly transferred to cavity B (Figure 7E). Thereby, coherences could be transferred spatially among the vibrational polaritons on the checkerboard cavity.

Interestingly, this transfer did not always occur. For example, when |UP_B_⟩⟨LP_B_| coherence was prepared in cavity B, there was no such transfer to cavity A (Figure 7C, bottom). The unidirectional coherence transfer suggested non-Hermitian dynamics. The reason for non-Hermitian dynamics was that at the same in-plane momentum, the photons that drove the coherence transfer always had higher energy when residing in cavity A than in cavity B. Thus, it was energetically favorable for photons from A to scatter to the state of cavity B at higher momentum without any energy penalty (Figure 7F). However, the same could not happen to photons at cavity B near zero in-plane momentum. When the same experiments occurred at high momentum, the coherence transfer became allowed in both directions (see the Supporting Information of ref (76)).

## Outlook and Key Future Challenges

Polaritons, a concept from quantum electrodynamics, have great potential to add a new paradigm to chemistry. Currently, many results have phenomenologically demonstrated the feasibility of this direction. It is critical to support this new phenomenon with a solid theoretical and mechanistic foundation. The works from our group used ultrafast 2D IR spectroscopy to follow the vibrational energy evolution inside VSC systems in a state-resolved manner. The results provide insights into what polaritons can do: First, polaritons can open and promote vibrational energy transfers, either within or between molecules; second, polaritons can also modify the dynamics of single-step chemical transformations; finally, inside of VSC, the dark modes do not show clear evidence of changing chemical dynamics. The impact of these results includes that they are unified with many theoretical predictions and lead to rational directions to improve polariton chemistry.

However, all of the experiments discussed here used an external IR pulse to pump the polaritons, while other VSC-modified experiments were carried out under thermally activated conditions, namely, there was no external photon input. The results of ultrafast spectroscopy here could be connected to the thermally activated VSC reactions by estimating the population of thermally excited polariton states. However, it is expected that the population is too small and insufficient to explain the thermally activated VSC reactions. Further connections remain to be explored. Furthermore, the detuning dependence of polariton chemistry,^12^ another outstanding experimental observation, remains to be better understood to explain why the energy match between the zero in-plane momentum cavity mode and vibrational modes determined the polariton chemistry; in contrast, cavity modes at higher momentum do not seem to matter. The recently developed zero-dispersion polariton modes in a confined cavity^79^ could further provide insight into the role of the dispersion curve in VSC-modified chemistry.

On the quantum technology side, we showed that polaritons can interact nonlinearly across space, similar to what is demonstrated in trapped ions or cold atoms, but under ambient conditions. It is natural to extend the checkerboard systems into a more sophisticated design to simulate natural systems with energy and coherence transfers or to achieve quantum effects, such as topological states.^80^ Obviously, the roadblock is decoherence. Fortunately, the vibrational coherence oscillates at the femtosecond time scale, which requires a much shorter coherence lifetime to achieve the same figure of merit of existing quantum systems. Decoherence could be alleviated by using molecular crystals or operating at a lower temperature (above cryogenic) where bath motions are slowed down or frozen. Another important development would be achieving VSC using nanocavities, which could significantly reduce the number of molecules needed for MVP formation and amplify the quantum features of the systems. Together with these developments, I expect that MVP could complement existing quantum information platforms.

Overall, molecular vibrational polaritons are an exciting field that integrates photonics into molecular science. The fundamental chemical physics of MVPs is critical to make this field a potential fast lane to advance chemistry and quantum technology.
